# Case Report: Liangxue Jiedu Huayu Formula as adjunctive therapy for leptospirosis with Weil's syndrome

**DOI:** 10.3389/fmed.2026.1905508

**Published:** 2026-10-08

**Authors:** Meixuan Li, Wenbin Huang, Mian Zhao, Qiao Zhuang, Xue Zhang, Yunan Wu

**Affiliations:** 1 Department of Infectious Diseases, The People’s Hospital of Xiangtan County, Xiangtan, China; 2 Department of Radiology, The People’s Hospital of Xiangtan County, Xiangtan, China; 3 Department of Hepatology/Infectious Diseases, The First Hospital of Hunan University of Chinese Medicine, Changsha, China; 4 Hunan University of Chinese Medicine, Changsha, China

**Keywords:** case report, leptospirosis, Liangxue Jiedu Huayu Formula, multiple organ dysfunction syndrome, Weil's syndrome

## Abstract

**Background:**

Leptospirosis is a global zoonotic disease with over one million cases annually worldwide. Approximately 5%–10% of patients progress to Weil's syndrome, characterized by multi-organ failure and high mortality, but treatment options remain limited. This report describes a patient with severe leptospirosis who showed marked improvement following treatment with Liangxue Jiedu Huayu Formula (LXJDHYF), a traditional Chinese medicine formula, and explores potential mechanisms.

**Case presentation:**

A 54-year-old male farmer developed high fever, jaundice, acute kidney injury, and early disseminated intravascular coagulation after working in a lotus pond. Leptospirosis was confirmed by a four-fold rise in MAT titer from 1:50 to 1:200. Despite initial treatment with conventional therapies including antibiotics, continuous renal replacement therapy, and plasma infusion, the patient remained febrile with persistently elevated white blood cell and bilirubin levels. From day 5 of hospitalization, LXJDHYF was added. Fever gradually subsided, total bilirubin progressively decreased, serum creatinine eventually normalized, and coagulation function returned to normal. At 6-month follow-up, the patient had fully recovered.

**Mechanistic exploration:**

Network pharmacology identified 14 common targets, including ICAM1, SELE, TLR4, and SERPINE1. KEGG enrichment revealed pathways related to TLR4/NF-*κ*B signaling, cytokine-cytokine receptor interaction, and complement/coagulation cascades. Molecular docking showed strong binding affinities (≤ −7.0 kcal/mol) between core components (cryptotanshinone, nortanshinone, rhein, naringenin) and core targets (TLR4, ICAM1, ALB). These computational findings are hypothesis-generating and require experimental validation.

**Conclusion:**

This case suggests that adjuvant treatment with LXJDHYF may be beneficial for leptospirosis complicated by Weil's syndrome, based on conventional therapy. Network pharmacology has generated testable hypotheses regarding multi-target modulation of inflammatory, adhesion, and coagulation pathways. These computational findings require experimental validation.

## 1 Introduction

Leptospirosis is acquired through percutaneous or mucosal contact with contaminated water or soil by pathogenic Leptospira and is highly regional in tropical and subtropical. In a few cases, this infection progresses to Weil's syndrome, which is characterized by jaundice, acute kidney injury, pulmonary hemorrhage and a high mortality rate (1). Severe leptospirosis is related to the dysregulated immune response (2). This endothelial injury triggers an inflammatory cascade via Toll-like receptor 2 and 4 recognition of leptospiral lipopolysaccharide and lipoproteins, activating innate immunity (3). Although antibiotic therapy plays a well-established role in the eradication of Leptospira, its clinical limitations have garnered increasing attention. Once the disease progresses to the immune phase, with complications such as pulmonary hemorrhage and renal failure, the therapeutic efficacy of antibiotics declines substantially (4). Corticosteroids may help attenuate the inflammatory response, but current evidence remains insufficient (5).

Leptospirosis is classified as a type of “warm disease” in traditional Chinese medicine (TCM), which is caused by exposure to “pestilential water”. Severe cases with jaundice are termed “Fulminant Jaundice”. The LXJDHYF was derived from the Xijiao Dihuang Formula and the Yinchenhao Formula, both with nearly two millennia of clinical use. The Xijiao Dihuang Formula, first recorded in Sun Simiao's Qianjin Fang [Sun (6), c. 652/2017], treats high fever-induced hemorrhage. The Yinchenhao Formula, from Zhang Zhongjing's Shanghan Lun [Zhang et al. (7), c. 220/2022], treats jaundice. Modern pharmacological investigations have substantiated that these Chinese medicinal agents exert pronounced therapeutic effects through multiple mechanisms, including anti-inflammatory activity (8), attenuation of endotoxin-induced injury (9), amelioration of cholestasis (10), and improvement of hepatic microcirculation (11).

Here we report a case of severe leptospirosis with multi-organ dysfunction syndrome (MODS) that responded to adjunctive LXJDHYF after conventional therapy showed limited efficacy. Given the absence of prior mechanistic studies on this formula in leptospirosis, we employed network pharmacology and molecular docking to generate hypotheses regarding its potential mechanisms.

## 2 Case presentation

Patient information has been de-identified to protect anonymity.

Patient Information and Presentation: A 54-year-old male patient as a farmer developed high fever six days before admission after working in a lotus pond. The patient had no significant past medical history, including no hypertension, diabetes, liver disease, or kidney disease. Family history was unremarkable for hereditary or infectious diseases. The patient has no history of smoking and occasionally drinks alcohol. Antibiotic treatment with intravenous piperacillin-tazobactam (4.5 g every 8 h) at a local primary hospital resulted in temporary defervescence, and no Jarisch–Herxheimer reaction was observed. Marked conjunctival injection was noted. However, liver and kidney function continued to deteriorate. Upon transfer to our hospital, the patient's total bilirubin exceeded 500 µmol/L. Chest and abdominal CT upon admission revealed extensive patchy opacities in both lungs, ground-glass infiltrates, and interstitial changes, with a mass-like and patchy nodular lesions in the right upper lobe. There was mild mucus plugging in the left main bronchus and segmental bronchi of the left lower lobe. The gallbladder showed heterogeneous density, suggestive of bile stasis and possible bile salt deposition. Both kidneys were enlarged, particularly the left kidney, raising suspicion of enlargement, along with mild parenchymal infiltration and perirenal fascial thickening. The sigmoid colon was elongated.

Diagnostic challenges: Initial MAT testing at the referring primary hospital was negative at a titer of 1:50 (institutional cutoff for positivity ≥1:100), delaying diagnosis. One week after admission, convalescent-phase serum testing at the local Center for Disease Control and Prevention (CDC) revealed seroconversion with a four-fold rise in titer to 1:200, confirming the diagnosis of leptospirosis according to WHO criteria. Differential diagnoses included viral hepatitis, acute cholangitis, sepsis from other bacterial sources, and hantavirus infection, all of which were ruled out based on serological and imaging findings.

Therapeutic Intervention: The patient initially received conventional supportive care, including antibiotics (intravenous ceftriaxone 2 g once daily for 14 days), methylprednisolone (40 mg once daily on Day 1 only), hepatoprotective agents, plasma transfusion, intermittent high-flow nasal cannula (HFNC) oxygen therapy, and continuous renal replacement therapy (CRRT). CRRT was administered in continuous venovenous hemodiafiltration (CVVHDF) mode with a blood flow rate of 150 mL/min, dialysate flow rate of 25 mL/kg/h, and replacement flow rate of 20 mL/kg/h, using regional citrate anticoagulation (target ionized calcium 0.25–0.35 mmol/L). Two sessions were performed: the first from 18:05 on Day 1 to 00:10 on Day 2 (approximately 6 h), and the second from 13:30 to 21:30 on Day 3 (8 h), totaling approximately 14 h of CRRT. Each session was performed in a general ward of the Department of Infectious Diseases, with continuous bedside monitoring by two dedicated dialysis nurses, and electrolyte and blood gas levels were monitored throughout the procedure. In addition, fresh frozen plasma (200 mL per unit) was infused on 8 separate occasions during the first 10 days of hospitalization (total 1,600 mL), and human albumin was administered on 4 separate occasions. The patient could not afford ICU care or additional CRRT sessions (each costing approximately USD 420); consequently, CRRT was discontinued after two sessions. Given the severity of the patient's multiorgan dysfunction syndrome, transfer to the ICU for advanced life support was strongly recommended. However, the patient was unable to afford the daily cost of ICU care and therefore remained in the general ward throughout the entire hospitalization. Serum creatinine declined markedly during the two CRRT sessions, from 780.5 µmol/L on admission to 255.0 µmol/L on day 4 (a 67.4% reduction), indicating that CRRT was the primary driver of early renal recovery. However, the patient remained febrile with the white blood cell count and the inflammatory marker procalcitonin continued to rise, clinical symptoms did not improve significantly, bilirubin levels failed to decrease progressively, and subcutaneous hemorrhage with early disseminated intravascular coagulation (DIC) developed as assessed by the Sepsis-Induced Coagulopathy (SIC) score. On day 5, LXJDHYF was added to the treatment. The formula consisted of: Paeonia lactiflora Pall. (Chishao) 30 g, Artemisia capillaris Thunb. (Yinchen) 30 g, Salvia miltiorrhiza Bunge. (Danshen) 15 g, Reynoutria japonica Houtt. (Huzhang) 15 g, Gardenia jasminoides J.Ellis. (Zhizi) 10 g, Rheum palmatum L. (Dahuang) 10 g, Paeonia suffruticosa Andr. (Mudanpi) 10 g, Bubalus bubalis L. (Shuiniujiao) 10 g, Citrus reticulata Blanco. (Chenpi) 10 g, and Atractylodes macrocephala Koidz. (Baizhu) 15 g. The herbs listed above represent one dose which was prepared according to the standard operating procedures of the hospital pharmacy.

On day 5, prior to the addition of LXJDHYF, the patient's SOFA score was 9 (improved from 13 on admission), and the SIC score was 3 (improved from 4 on day 3). Despite some improvement in renal function following CRRT and partial improvement in coagulation parameters, the patient remained febrile (38.6 °C) with elevated inflammatory markers (white blood cell count 28.0 × 10^9^/L). These findings indicated that although supportive therapies had partially stabilized organ function, the underlying inflammatory response and hepatic injury persisted, prompting the addition of LXJDHYF.

Follow-up and Outcomes: After the addition of LXJDHYF, the patient's bowel movements regularized, fever subsided, and laboratory parameters improved markedly and progressively. One week post-admission, both leptospirosis IgM and IgG were positive. No adverse events occurred. The local Center for Disease Control and Prevention reported that the patient's single serum sample tested positive for leptospirosis by microscopic agglutination test (MAT) at a titer of 1:200. The patient adhered to the prescribed LXJDHYF regimen without missing any doses. On day 8, the LXJDHYF formula was adjusted by increasing Chishao and decreasing Yinchen to enhance blood circulation as jaundice began to resolve; on day 15, Chishao and Dahuang were reduced to prevent excessive purgation after the patient developed loose stools. No adverse events or unexpected reactions were observed during the treatment period. At 3 months, all findings had normalized; at 6 months, the patient had fully resumed physical labor (Table 1, Figure 1).

**Table 1:** Clinical course and laboratory parameters during hospitalization.

Day	Symptoms & Signs	T (°C)	Stool	WBC (×10^9^/L)	PLT (×10^9^/L)	Cr (µmol/L)	TBIL (µmol/L)	DBIL (µmol/L)	TBA (µmol/L)	APTT (s)	SOFA/SIC	FIB (g/L)	Key Interventions
1	Jaundice, fatigue, hemoptysis	37.3	−	14.4	55	780.5	519.1	352.4	310.1	Normal	13/4	5.1	Ceftriaxone 2 g QD; methylprednisolone; CRRT (18:05-Day 2 00:10); plasma transfusion (200 mL)
2	Jaundice, fatigue, hemoptysis, SpO₂ drop	37.3	−	14.0	63	493.2	529.0	343.2	233.6	Normal	−	4.2	No CRRT; HFNC added;plasma transfusion (200 mL)
3	Jaundice, fatigue, hemoptysis, fever (new onset)	38.5	−	19.9	105	384.5	564.1	353.7	305.2	45.9	NC/4	Normal	CRRT (13:30–21:30)
4	Jaundice, persistent fever, petechiae, no hemoptysis	38.5	−	27.1	110	255.0	487.8	313.0	159.6	56.6	−	4.2	plasma transfusion (200 mL)
5	Jaundice, fever, petechiae	39.3	−	28.0	Normal	258.2	443.1	293.0	133.5	63.7	9/3	4.8	**LXJDHYF initiated;**plasma transfusion (200 mL);
6	Fever persists, petechiae reduced, bowel movement returns	38.0	−	24.5	Normal	204.3	424.3	277.7	136.8	60.9	−	4.8	LXJDHYF continued;plasma transfusion (200 mL);
7	Fever persists, petechiae resolved, normal bowel movement	38.6	+	23.5	Normal	164.2	355.6	236.6	158.8	54.7	−	4.5	LXJDHYF continued;plasma transfusion (200 mL);Electrolyte correction;
8	Moderate jaundice, improving appetite	38.4	+	21.9	Normal	139.5	257.9	176.2	106.2	48.9	−	4.2	LXJDHYF adjusted (↑ Chishao, ↓ Yinchen);plasma transfusion (200 mL)
10	Moderate jaundice, improving appetite	36.5	+	11.3	Normal	110.1	129.3	87.9	20.9	Normal	−	Normal	LXJDHYF continued;plasma transfusion (200 mL)
15	Mild jaundice	36.8	+	9.6	Normal	87.4	80.6	53.9	4.1	46.8	3/0	Normal	LXJDHYF adjusted (↓ Chishao, ↓ Dahuang)
25	Mild jaundice	36.4	+	7.7	Normal	81.9	45.9	29.0	8.2	Normal		Normal	Follow-up; continued recovery

WBC, white blood cell count; Cr, creatinine; TBIL, total bilirubin; DBIL, direct bilirubin; TBA, total bile acid; APTT, activated partial thromboplastin time; FIB, fibrinogen; CRRT, continuous renal replacement therapy; HFNC, high-flow nasal cannula; LXJDHYF, Liangxue Jiedu Huayu Formula; QD, once daily. “−” indicates not tested or not applicable.

^a^Unless otherwise specified in the “Key Interventions” column, all ongoing treatments (including ceftriaxone 2 g QD and supportive care) were continued on the days without specific notation.

^b^SOFA and SIC scores were assessed at admission (Day 1: SOFA 13, SIC 4), Day 3 (SIC 4; SOFA NC due to missing PaO₂/FiO₂ data), Day 5 (SOFA 9, SIC 3), and discharge (SOFA 3, SIC 0). “−” indicates not assessed on other days; NC indicates not calculable.

**Figure 1:**
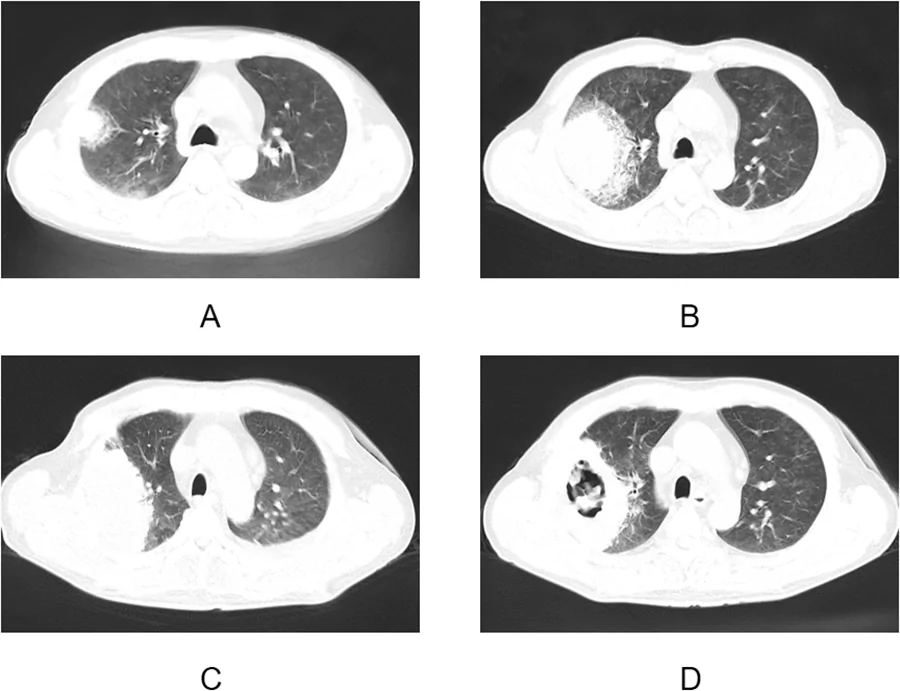
Serial chest CT images (lung window) of the patient with leptospirosis on day 1 **(A)**, day 4 **(B)**, day 10 **(C)**, and day 25 **(D)** after admission. **(A)** Day 1: Bilateral widespread patchy and ground-glass opacities with interstitial changes. A right upper lobe mass with patchy nodular opacities. Minimal mucus plugs in the left bronchus and left lower lobe segmental bronchi. **(B)** Day 4: Increased bilateral patchy exudative opacities. Enlarged right upper lobe mass. Mild bilateral pleural effusion with mild atelectasis. **(C)** Day 10: Decreased bilateral patchy exudative opacities. Further enlarged right upper lobe mass. Bilateral lower lobe atelectasis. Increased bilateral pleural effusion. **(D)** Day 25: Decreased size of right upper lobe mass with new cavitation and surrounding new inflammatory changes. Nearly resolved minimal inflammation in both lower lobes. Nearly resolved minimal bilateral pleural effusion.

## 3 Methods

### 3.1 Herbal preparation

The LXJDHYF were decocted by the standard operating procedures of the hospital pharmacy: each dose was soaked in 1 L of pure water for 30 min, then boiled for another 30 min, and the filtrate was collected; the medicinal residue was re-added with 1 L of pure water, sealed and boiled for 20 min. The filtrate from the two times was mixed and concentrated to approximately 300 mL, which was to be taken orally within one day, divided into two warm doses (40 °C). All medicinal for formula pieces were identified in accordance with the Chinese Pharmacopoeia (Chinese Pharmacopoeia Commission, 2020), and the dosage was determined based on classic prescriptions and modern pharmacological literature. This detailed plan was provided to ensure repeatability. No voucher specimens were retained for the clinical finished herbal pieces.

### 3.2 Screening of active components and target prediction

The main ingredients of LXJDHYF: Baizhu, Chenpi, Chishao, Dahuang, Danshen, Huzhang, Mudanpi, Shuiniujiao, Yinchen, Zhizi—were screened for active compounds using the TCM Systems Pharmacology (TCMSP) database (version 2.3) with the following parameters: oral bioavailability (OB) ≥ 30% and drug-likeness (DL) ≥ 0.18. Potential target proteins were then obtained and validated through a conversion process using the PubChem database, the National Center for Biotechnology Information (NCBI) database (Release 235), and the SwissTargetPrediction database. Subsequently, the UniProt protein database (release 2026_02) was employed to convert the target proteins into unified gene names.

### 3.3 Identification and network visualization of drug-disease common targets (DDT)

To identify potential therapeutic targets, disease-associated genes were retrieved from the GeneCards (version 5.21), OMIM, and DisGeNET databases (version 7.0) using “Leptospira” and “Leptospirosis” as search keywords. The Interactive Venn platform was employed to identify the overlapping targets between the active components of the herbal formula and the disease-related genes. The resulting set of common targets was designated as drug-disease common targets. Subsequently, a drug-disease network was constructed and visualized using Cytoscape software (version 3.7.1).

### 3.4 Protein-Protein interaction (PPI) network construction

The DDT results were submitted to the STRING database (version 12.5) for PPI network construction, restricted to Homo sapiens, with a minimum interaction score threshold of 0.700. The PPI network was visualized using Cytoscape software (version 3.7.1).

### 3.5 GO functional enrichment analysis and KEGG pathway enrichment analysis

The DDT results were submitted to the Metascape database (version 3.5) for GO Functional Enrichment Analysis and to the KOBAS platform (v3.0.3) for KEGG Pathway Enrichment Analysis. The result data were visualized using the SRplot platform and Cytoscape software (Figure 2).

**Figure 2:**
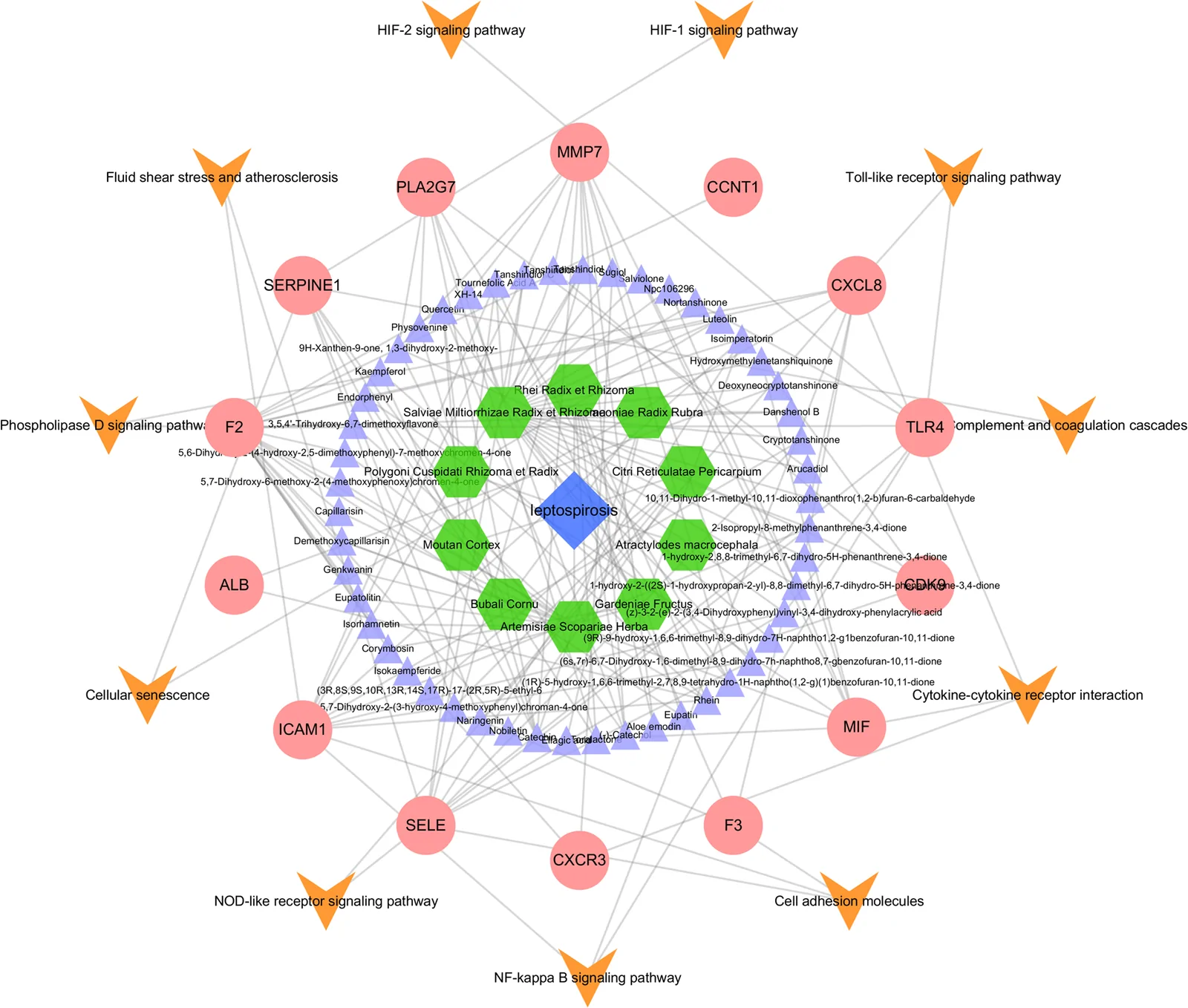
Schematic diagram of the “disease–herb–component–target–pathway” association network. Different shapes and colors denote distinct entity types: blue diamonds represent disease names, green hexagons represent herb names, lavender triangles represent herb components, peach circles represent disease targets, and yellow V-shapes represent signaling pathways. Lines indicate the interrelationships among entities.

### 3.6 Molecular docking validation

To validate the binding affinity between core active compounds and core target proteins, molecular docking simulations were performed using AutoDock Vina software (version 1.5.7). Protein preparation: The three-dimensional structures of the core target proteins (TLR4, ICAM1, SELE, SERPINE1 and ALB) were retrieved from the RCSB Protein Data Bank (PDB), respectively. Protein structures were processed using AutoDock Tools (version 1.5.7): water molecules and co-crystallized ligands (except for the reference ligand) were removed, polar hydrogens were added, and charges were assigned. Ligand preparation: The three-dimensional structures of the core active compounds (cryptotanshinone, nortanshinone, rhein, Naringenin.) were generated from their SMILES strings using Open Babel software (version 3.1.0) and energy-minimized using the MMFF94 force field. Docking parameters: Grid box center coordinates and dimensions were set according to the active binding sites of each target protein. Fifty independent docking runs were performed for each ligand-target pair. All other parameters were set to AutoDock Vina default values. Docking results were reported as binding affinity (kcal/mol). A binding energy ≤ −7.0 kcal/mol was defined as the threshold for strong binding interactions, based on previously published literature (12). The validity of the docking protocol was verified by re-docking the co-crystallized ligand into the binding pocket, with an RMSD < 2.5 Å considered acceptable (Figure 3).

**Figure 3:**
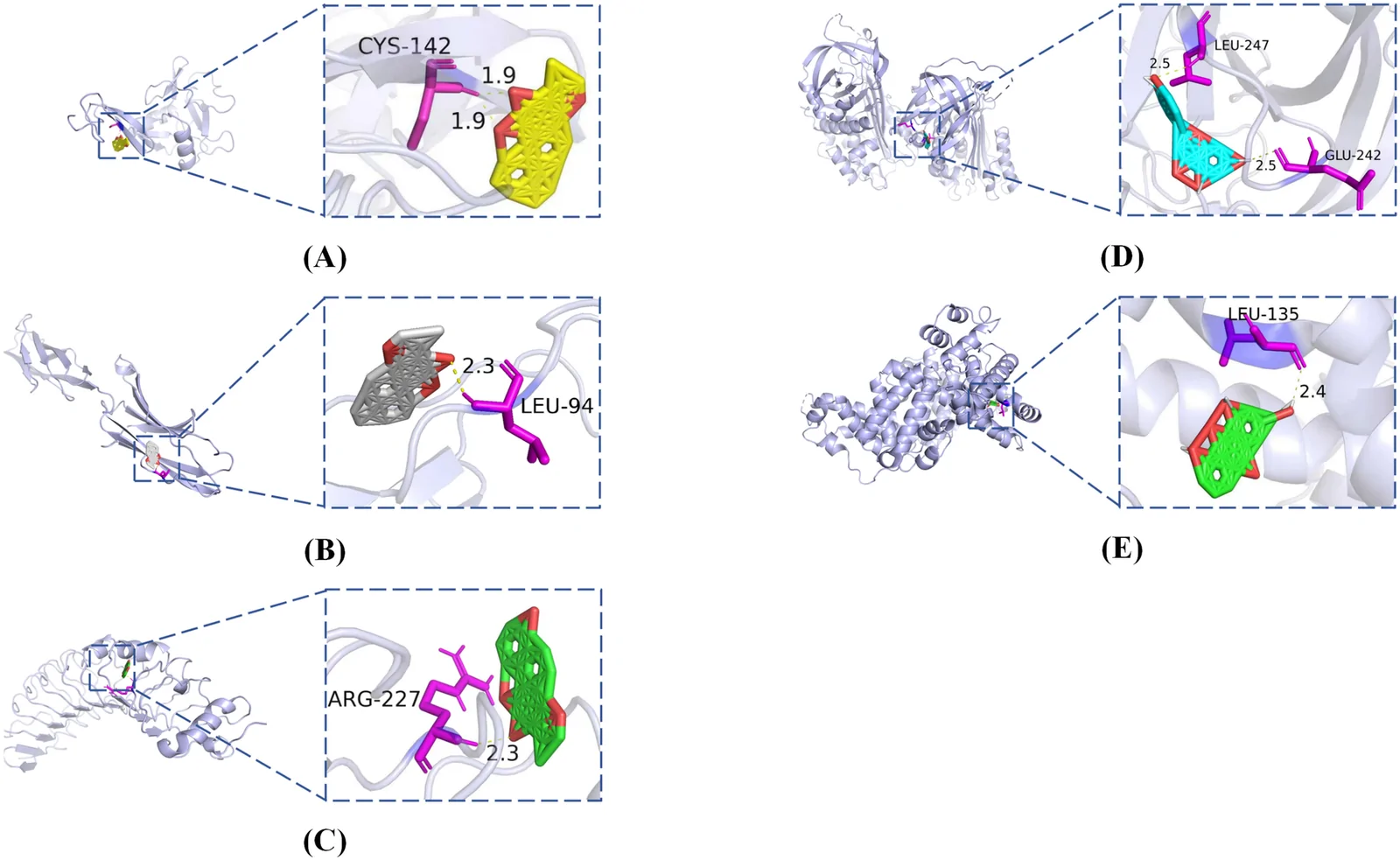
Molecular docking results. **(A–E)** For each panel, the left side shows the overall docking complex, with a dashed square highlighting the detailed binding site, and the right side provides a magnified view of the interaction details. Yellow dashed lines indicate hydrogen bonds, with bond lengths labeled in angstroms **(Å)** The calculated binding energies are as follows: **(A)** −7.67 kcal/mol, **(B)** −7.23 kcal/mol, **(C)** −7.82 kcal/mol, **(D)** −5.34 kcal/mol, and **(E)** −7.08 kcal/mol. Cryptotanshinone formed a hydrogen bond with ICAM-1 at Leu94 (2.3 Å) and two hydrogen bonds with Cys142 (1.9 Å). Nordanshengin bound to TLR4 at Arg227 (2.3 Å). Rhein interacts with albumin (ALB) at the Leu135 site (2.4 Å). Naringenin formed two hydrogen bonds (2.5 Å each) with SERPINE1.

### 3.7 Limitations of the computational analyses

The network pharmacology and molecular docking results are computational predictions and hypothesis-generating in nature. Key limitations include: (1) OB/DL screening does not reflect actual *in vivo* bioavailability; (2) numerous unanalyzed components in the formula may also contribute to activity; (3) synergistic/antagonistic interactions between components are not captured; (4) docking scores do not equate to biological activity; and (5) these mechanistic hypotheses require experimental validation through *in vitro*, cellular, and *in vivo* studies.

## 4 Discussion

### 4.1 TCM syndrome differentiation

This case demonstrates the potential benefits of combining conventional Western medicine with LXJDHYF in the treatment of leptospirosis with Weil's Syndrome. According to TCM principles, the patient presented with fever, subcutaneous petechiae, elevated inflammatory indicators, generalized jaundice, constipation, which were consistent with the “heat entering the blood camp syndrome”, that is, “damp-heat toxins stagnate in the liver and gallbladder”. The formula's design reflects this differentiation: Chishao, Yinchen cool blood and resolve jaundice; Huzhang, Zhizi, Dahuang clear heat and promote bile excretion; Danshen, Mudanpi, Shuiniujiao for removing blood stasis and protecting the endothelium.

### 4.2 Potential mechanisms

Antipyretic and anti-inflammatory effects: Danshen contains diterpenoids and phenolic acids, which have been proven to have excellent anti-inflammatory effects, and also exert good regulatory effects on TNF-α and IL-1β (13); The compounds such as quercetin, kaempferol and baicalein in Mudanpi have a good anti-inflammatory effect and can regulate the NF-κB pathway (14). The sulfhydryl-rich peptide fractions (SHPF) contained in the horn keratin of Shuiniujiao can inhibit the inflammatory pathways and reduce inflammatory factors, especially showing excellent antipyretic effects (15).

Hepatoprotective and bile acid-lowering effects: The Yinchen-Zhizi-Dahuang triad enhances bilirubin conjugation and promotes bile secretion, including important compounds such as chlorogenic acid, geniposide, and rhein (16, 17).

Renal protection and potential supportive role: Anthraquinone in Dahuang can alleviate pathological damage to kidney tissues (18). Danshen-Shaoyao can improve the thrombotic state by up-regulating the expression of EGFR mRNA and inhibiting the expression of F10 and SRC mRNA. F10 is a key factor in the coagulation cascade reaction, and its inhibition can directly reduce fibrin production and thrombosis (19, 20).

Laxative effect to expel pathogenic heat: Dahuang unblocks the bowels to expel pathogenic heat. Its anthraquinone components increase intestinal permeability, allowing toxin and bilirubin excretion through defecation, and reducing portal endotoxin load via the “gut-liver axis” (21, 22).

Improvement of microcirculation and correction of coagulopathy: Stasis-resolving components (Chishao, Danshen, Mudanpi) and compounds like paeoniflorin,which play a significant role in inhibiting abnormal platelet aggregation and improve microcirculation (23, 24). In shuiniujiao, Thiols-rich peptide fractions have a protective effect on oxidative damage to vascular endothelial cells (25). This “bidirectional regulatory” effect resolves the paradoxical thrombosis-hemorrhage dilemma in DIC (26, 27).

### 4.3 Network pharmacology insights

The identification of TLR4, ICAM1, SELE and SERPINE1 as core targets is highly consistent with the pathophysiology of leptospiral disease—inflammatory storm, endothelial injury and coagulation dysfunction. KEGG enrichment—particularly the TLR4/NF-*κ*B signaling and complement/coagulation cascades—precisely matches the pathological process of severe leptospirosis. Molecular docking provides structural support for the interactions of active components such as geniposide, neodammoniacoside, and naringenin with these targets, suggesting a “multiple-site blocking” regulatory mode.

### 4.4 Comparison and strengths/limitations

This case demonstrates TCM's potential as an effective, low-cost adjunct when conventional therapy fails. Strengths include detailed longitudinal data, mechanistic exploration, and long-term follow-up. We acknowledge that the patient received multiple concurrent interventions (antibiotics, CRRT, plasma infusion, and supportive therapies), and the observed clinical improvement cannot be attributed solely to LXJDHYF. Limitations are its single-case nature, computational predictions, and the inability to isolate independent effect of LXJDHYF.

### 4.5 Take-away lessons

This case provides several important lessons for managing severe leptospirosis, particularly in resource-limited settings. First, this case demonstrates that LXJDHYF may represent a potentially useful adjunctive option when conventional therapy is insufficient. Second, the coagulation profile in this case presented a complex and dynamic picture. Notably, although the platelet count had returned to normal levels by day 5 with supportive therapy, the patient subsequently developed subcutaneous petechiae, along with progressively prolonged APTT and decreased fibrinogen levels, indicating an ongoing consumption coagulopathy. Following the addition of LXJDHYF, coagulation parameters gradually stabilized and the skin petechiae resolved. This temporal sequence raises the possibility that LXJDHYF may have contributed to restoring hemostatic balance. This hypothesis is supported by recent literature documenting pro-fibrinolytic and endothelial-protective effects of its constituent herbs [Rao et al. (23); Shang et al. (27)]. However, given the confounding effects of plasma transfusions and other supportive measures, this observation remains hypothesis-generating and requires further validation. Third, the low cost of LXJDHYF (∼0.55 USD/day) improved adherence in this patient who had considered abandoning therapy, suggesting affordability as a practical advantage in resource-limited settings, though formal cost-effectiveness analysis is needed. Fourth, network pharmacology and molecular docking analyses have identified potential multi-target mechanisms involving TLR4/NF-κB signaling, inflammatory adhesion molecules, and coagulation pathways, suggesting a possible molecular basis that warrants experimental investigation. Together, these findings suggest that early adjunctive LXJDHYF therapy, when integrated with conventional treatment, may help break the cycle of progressive organ failure in Weil's syndrome and warrants further investigation in larger studies.

### 4.6 Clinical and research implications

Despite its inherent limitations, this case offers several insights for clinical practice and future research. First, there was a temporal correlation between the addition of LXJDHYF and the reversal of clinical deterioration. This was particularly evident in the simultaneous improvement of fever, leukocytosis, and hyperbilirubinemia, suggesting that this formula may act through multiple target points to address the core pathophysiological processes of Weil's syndrome, namely systemic inflammation, endothelial injury, and coagulation dysfunction. This is consistent with the results predicted by network pharmacology that LXJDHYF targets multiple pathways (including the TLR4/NF-κB signaling pathway, cytokine-cytokine receptor interaction, and complement/coagulation cascade reaction). Second, from a clinical perspective, LXJDHYF is inexpensive (about $0.55 per day), making it a potentially attractive adjunctive treatment option in regions with limited resources and difficult access to intensive care. Third, from a research perspective, the computational predictions generated in this study provide a clear roadmap for future experimental verification: the interactions between key compounds (cryptotanshinone, nortanshinone, rhein, naringenin) and core targets (TLR4, ICAM1, ALB) should be verified *in vitro*, while the therapeutic effect of LXJDHYF should be evaluated in a leptospiral disease animal model before a definite conclusion can be drawn about its mechanism of action. Finally, this case indicates that combining traditional Chinese medicine with conventional treatment may provide a new approach for the treatment of severe leptospirosis, but this assumption needs to be rigorously verified in larger-scale controlled studies.

## 5 Patient perspective

The patient reported that after starting to use LXJDHYF, they felt “significantly improved,” with fatigue reduced, fever subsided, appetite restored, and intestinal function returned to normal. Due to financial constraints, the patient was unable to afford the cost of being transferred to the intensive care unit for advanced life support, and the cost of continuous renal replacement therapy (CRRT) was also prohibitive. At one point, they even considered giving up the treatment. The LXJDHYF plan costs 4 yuan per day (approximately 0.55 US dollars), which significantly reduces his financial burden. At the same time, it promotes clinical recovery within a short period of time. The patient was very satisfied with the treatment and expressed a willingness to recommend this method to other patients with similar conditions.

## Data Availability

The datasets presented in this study can be found in online repositories. The names of the repository/repositories and accession number(s) can be found in the article/Supplementary Material.
