# Supplementary material for: Case Report: Liangxue Jiedu Huayu Formula as adjunctive therapy for leptospirosis with Weil's syndrome
Source: Front Med (Lausanne). 2026 Oct 8;13:1905508. doi: 10.3389/fmed.2026.1905508 (PMC13686832; doi:10.3389/fmed.2026.1905508)
Supplement: Supplementary file 1 [file Table1.docx]

Case Report: Liangxue Jiedu Huayu Formula as Adjunctive Therapy for Leptospirosis with Weil’s Syndrome

# 1 Supplementary Table

| **Day** | **Symptoms & Signs** | **T (°C)** | **Stool** | **WBC (×10⁹/L)** | **PLT (×10⁹/L)** | **Cr (µmol/L)** | **TBIL (µmol/L)** | **DBIL (µmol/L)** | **TBA (µmol/L)** | **APTT (s)** | **SOFA/SIC** |  | **FIB (g/L)** | **Key Interventions** |
| --- | --- | --- | --- | --- | --- | --- | --- | --- | --- | --- | --- | --- | --- | --- |
| 1 | Jaundice, fatigue, hemoptysis | 37.3 | - | 14.4 | 55 | 780.5 | 519.1 | 352.4 | 310.1 | Normal | 13 / 4 |  | 5.1 | Ceftriaxone 2 g QD; methylprednisolone; CRRT (18:05-Day 2 00:10); plasma transfusion (200 mL) |
| 2 | Jaundice, fatigue, hemoptysis, SpO₂ drop | 37.3 | - | 14.0 | 63 | 493.2 | 529.0 | 343.2 | 233.6 | Normal | - |  | 4.2 | No CRRT; HFNC added;plasma transfusion (200 mL) |
| 3 | Jaundice, fatigue, hemoptysis, fever (new onset) | 38.5 | - | 19.9 | 105 | 384.5 | 564.1 | 353.7 | 305.2 | 45.9 | NC / 4 |  | Normal | CRRT (13:30-21:30) |
| 4 | Jaundice, persistent fever, petechiae, no hemoptysis | 38.5 | - | 27.1 | 110 | 255.0 | 487.8 | 313.0 | 159.6 | 56.6 | - |  | 4.2 | plasma transfusion (200 mL) |
| **5** | Jaundice, fever, petechiae | 39.3 | - | 28.0 | Normal | 258.2 | 443.1 | 293.0 | 133.5 | 63.7 | 9 / 3 |  | 4.8 | **LXJDHYF initiated;**plasma transfusion (200 mL); |
| 6 | Fever persists, petechiae reduced, bowel movement returns | 38.0 | + | 24.5 | Normal | 204.3 | 424.3 | 277.7 | 136.8 | 60.9 | - |  | 4.8 | LXJDHYF continued;plasma transfusion (200 mL); |
| 7 | Fever persists, petechiae resolved, normal bowel movement | 38.6 | + | 23.5 | Normal | 164.2 | 355.6 | 236.6 | 158.8 | 54.7 | - |  | 4.5 | LXJDHYF continued;plasma transfusion (200 mL);Electrolyte correction; |
| 8 | Moderate jaundice, improving appetite | 38.4 | + | 21.9 | Normal | 139.5 | 257.9 | 176.2 | 106.2 | 48.9 | - |  | 4.2 | LXJDHYF adjusted (↑ Chishao, ↓ Yinchen);plasma transfusion (200 mL) |
| 10 | Moderate jaundice, improving appetite | 36.5 | + | 11.3 | Normal | 110.1 | 129.3 | 87.9 | 20.9 | Normal | - |  | Normal | LXJDHYF continued;plasma transfusion (200 mL) |
| 15 | Mild jaundice | 36.8 | + | 9.6 | Normal | 87.4 | 80.6 | 53.9 | 4.1 | 46.8 | 3 / 0 |  | Normal | LXJDHYF adjusted (↓ Chishao, ↓ Dahuang) |
| 25 | Mild jaundice | 36.4 | + | 7.7 | Normal | 81.9 | 45.9 | 29.0 | 8.2 | Normal |  |  | Normal | Follow-up; continued recovery |

Table 1 Clinical course and laboratory parameters during hospitalization

# Abbreviations: WBC, white blood cell count; Cr, creatinine; TBIL, total bilirubin; DBIL, direct bilirubin; TBA, total bile acid; APTT, activated partial thromboplastin time; FIB, fibrinogen; CRRT, continuous renal replacement therapy; HFNC, high-flow nasal cannula; LXJDHYF, Liangxue Jiedu Huayu Formula; QD, once daily. “−” indicates not tested or not applicable.

# † Unless otherwise specified in the “Key Interventions” column, all ongoing treatments (including ceftriaxone 2 g QD and supportive care) were continued on the days without specific notation.

# ‡ SOFA and SIC scores were assessed at admission (Day 1: SOFA 13, SIC 4), Day 3 (SIC 4; SOFA NC due to missing PaO₂/FiO₂ data), Day 5 (SOFA 9, SIC 3), and discharge (SOFA 3, SIC 0). “-” indicates not assessed on other days; NC indicates not calculable.

# 2 Supplementary Figure

**Figure 1.**Serial chest CT images (lung window) of the patient with leptospirosis on day 1 (A), day 4 (B), day 10 (C), and day 25 (D) after admission. (A) Day 1: Bilateral widespread patchy and ground-glass opacities with interstitial changes. A right upper lobe mass with patchy nodular opacities. Minimal mucus plugs in the left bronchus and left lower lobe segmental bronchi. (B) Day 4: Increased bilateral patchy exudative opacities. Enlarged right upper lobe mass. Mild bilateral pleural effusion with mild atelectasis. (C) Day 10: Decreased bilateral patchy exudative opacities. Further enlarged right upper lobe mass. Bilateral lower lobe atelectasis. Increased bilateral pleural effusion. (D) Day 25: Decreased size of right upper lobe mass with new cavitation and surrounding new inflammatory changes. Nearly resolved minimal inflammation in both lower lobes. Nearly resolved minimal bilateral pleural effusion.

**Figure 2.** Schematic diagram of the “disease–herb–component–target–pathway” association network. Different shapes and colors denote distinct entity types: blue diamonds represent disease names, green hexagons represent herb names, lavender triangles represent herb components, peach circles represent disease targets, and yellow V-shapes represent signaling pathways. Lines indicate the interrelationships among entities.

**Figure 3.**Molecular docking results. (A–E) For each panel, the left side shows the overall docking complex, with a dashed square highlighting the detailed binding site, and the right side provides a magnified view of the interaction details. Yellow dashed lines indicate hydrogen bonds, with bond lengths labeled in angstroms (Å). The calculated binding energies are as follows: (A) –7.67 kcal/mol, (B) –7.23 kcal/mol, (C) –7.82 kcal/mol, (D) –5.34 kcal/mol, and (E) –7.08 kcal/mol. Cryptotanshinone formed a hydrogen bond with ICAM-1 at Leu94 (2.3 Å) and two hydrogen bonds with Cys142 (1.9 Å). Nordanshengin bound to TLR4 at Arg227 (2.3 Å). Rhein interacts with albumin (ALB) at the Leu135 site (2.4 Å). Naringenin formed two hydrogen bonds (2.5 Å each) with SERPINE1.
